# Ciprofibrate-Loaded Nanoparticles Prepared by Nanoprecipitation: Synthesis, Characterization, and Drug Release

**DOI:** 10.3390/polym13183158

**Published:** 2021-09-18

**Authors:** Raissa Lohanna Gomes Quintino Corrêa, Renan dos Santos, Lindomar José Calumby Albuquerque, Gabriel Lima Barros de Araujo, Charlotte Jennifer Chante Edwards-Gayle, Fabio Furlan Ferreira, Fanny Nascimento Costa

**Affiliations:** 1 Center for Natural and Human Sciences (CCNH), Federal University of ABC (UFABC), Santo André 09210-580, Brazil; reeh_sants@hotmail.com (R.d.S.); lindomaralbuquerque@gmail.com (L.J.C.A.); fabio.furlan@ufabc.edu.br (F.F.F.); 2 Brazilian Synchrotron Light Laboratory (LNLS), Brazilian Center for Research in Energy and Materials (CNPEM), Campinas 13083-170, Brazil; 3 Department of Pharmacy, Faculty of Pharmaceutical Sciences, University of Sao Paulo, Sao Paulo 05508-900, Brazil; gabriel.araujo@usp.br; 4 Diamond Light Source, Harwell Science & Innovation Campus, Didcot, Oxfordshire OX11 0DE, UK; charlotte.edwards-gayle@diamond.ac.uk; 5 Nanomedicine Research Unit (NANOMED), Federal University of ABC (UFABC), Santo André 09210-580, Brazil

**Keywords:** ciprofibrate, drug delivery, Rietveld method, crystallography, nanotechnology

## Abstract

Ciprofibrate (CIP) is a highly lipophilic and poorly water-soluble drug, typically used for dyslipidemia treatment. Although it is already commercialized in capsules, no previous studies report its solid-state structure; thus, information about the correlation with its physicochemical properties is lacking. In parallel, recent studies have led to the improvement of drug administration, including encapsulation in polymeric nanoparticles (NPs). Here, we present CIP’s crystal structure determined by PXRD data. We also propose an encapsulation method for CIP in micelles produced from Pluronic P123/F127 and PEO-*b*-PCL, aiming to improve its solubility, hydrophilicity, and delivery. We determined the NPs’ physicochemical properties by DLS, SLS, ELS, SAXS and the loaded drug amount by UV-Vis spectroscopy. Micelles showed sizes around 10–20 nm for Pluronic and 35–45 nm for the PEO-*b*-PCL NPs with slightly negative surface charge and successful CIP loading, especially for the latter; a substantial reduction in ζ-potential may be evidenced. For Pluronic nanoparticles, we scanned different conditions for the CIP loading, and its encapsulation efficiency was reduced while the drug content increased in the nanoprecipitation protocol. We also performed in vitro release experiments; results demonstrate that probe release is driven by Fickian diffusion for the Pluronic NPs and a zero-order model for PEO-*b*-PCL NPs.

## 1. Introduction

Ciprofibrate (CIP), chemical formula C_13_H_14_C_l2_O_3_, is classified as a synthetic active pharmaceutical ingredient (API), which belongs to the fibrate class of drugs, generally used against dyslipidemia, a condition characterized by abnormal lipid levels in the blood system [1]. Dyslipidemia is a risk factor for developing cardiac diseases such as atherosclerosis, an inflammation characterized by the formation of fat, calcium, and other elements’ plates in the walls of the heart’s arteries and vascular system in general. Atherosclerosis can lead to different cardiac diseases responsible for more than 17.3 million deaths annually worldwide [2]. Nowadays, CIP is commercially available for oral formulations as tablets and capsules (100 mg) [3], and patients report several side effects such as headaches, nausea, and diarrhea [4].

In pharmaceutical development, there is considerable interest in crystal structure investigations, as a way to understand how their structures (either amorphous or crystalline) and properties such as density, size, and particle shape correlate to other physicochemical properties, aiming the optimization of these drugs for solid dosage [5].

A relevant characteristic of ciprofibrate is its hydrophobic behavior. Designated by the Biopharmaceutics Classification System (BCS) as a class II drug, CIP presents low solubility and high permeability [6]. The high permeability allows a complete absorption of the drug by the small intestine; otherwise, its poor solubility limits its application to treatments. One possible way to overcome this problem is the use of micro- or nanostructures for improved drug delivery.

Nanomedicine—an interdisciplinary area that merges nanotechnology and medicine—has investigated several solutions that reach more efficient treatments with minimum side effects for various diseases. Several types of nanocarriers have been developed; structures such as polymeric micelles, liposomes, and NP that conjugate with drugs by diverse mechanisms (encapsulation, surface adsorption, and others) are used to deliver controlled and localized drug dosages to the body, implementing the drug delivery concept [7,8,9,10]. Each of these techniques presents a myriad of controllable features that can influence important aspects of the nanostructures. Encapsulation methods, in particular, may vary among different combinations of hydrophilic and lipophilic block copolymers and steps for synthesis, changing size, loading capacity, and even drug release behavior [11,12].

The poloxamer surfactant (or Pluronic, known by its trade name) is one of the most extensively investigated biomaterials used to build nanocarriers. These are typical triblock copolymers comprising two unities of poly (ethylene oxide) (PEO) and one of poly (propylene oxide) (PPO) in an alternated linear structure. Although not classified as biodegradable, PEO and derivatives present many advantages for biomedical usage such as relative safety profile (lethal dosage being LD50s > 5 g kg^−1^), FDA approval, lack of immunogenicity, and particularly, the ease of excretion from living organisms [13]. The remaining parts of the molecules are found in living systems since they are aliphatic chains (derived from fatty acid) and sugar molecules that are dietary sources of fuel and important structural components of cells. Additionally, taking into account the molecular weight of the amphiphilic chains (PCL Mw 18,500 g mol^−1^, P123 Mw 5750 g mol^−1^, and F127 12,600 g mol^−1^), the materials can undergo renal clearance (Mw < 40,000 g mol^−1^) [13,14]. Their block copolymer structures can self-assemble in different structural forms such as micelles, worm-like micelles, or vesicles in the aqueous medium. This approach allows the encapsulation of hydrophobic drugs such as CIP, increasing their solubility, improving circulation in vivo, and avoiding aggregation problems [15].

**Figure 1:**
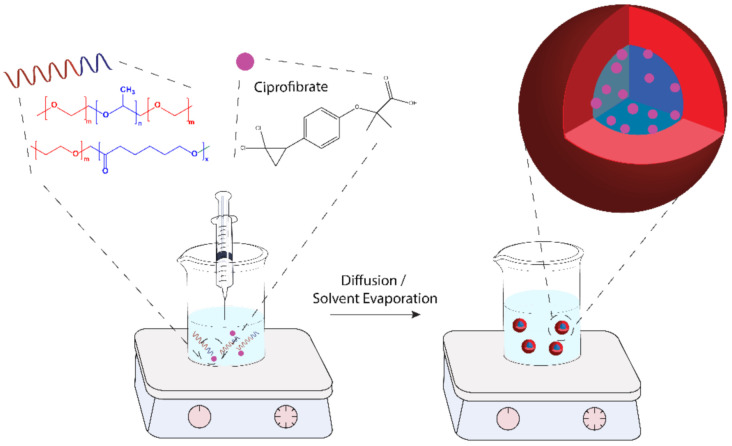
Scheme of preparation by nanoprecipitation of ciprofibrate-loaded nanoparticles.

Different types of Pluronic are used together as they present better properties such as colloidal stability and better drug-carrying efficiency [15], especially for the self-assembly into micelle morphology [16]. In this work, P123 and F127 block copolymers were used together (PEO_20_-*b*-PPO_70_-*b*-PEO_20_ and PEO_100_-*b*-PPO_65_-*b*-PEO_100_, respectively) to produce ciprofibrate-loaded micelles. F127 is a widely known and investigated copolymer for its thermoreversible gelation property at body temperature [17]. Another block copolymer was considered for preparing the CIP-loaded micelles: the PEO_113_-*b*-PCL_118_ was chosen due to its biocompatibility, biodegradability, and proven record in soft-based nanocarrier platforms for therapeutic applications [18,19]. The nanoprecipitation process was selected to prepare the CIP-loaded micelles, and an illustration of the method is provided in Figure 1. 

Recent results suggest that fenofibrate, which belongs to the same drug class as CIP, may have an essential role in controlling SARS-CoV-2 infection in in vitro models [20]. Considering that the reduction in serum triglycerides and LDL cholesterol has a positive impact in the fighting against numerous age-related diseases and also against the recent COVID-19 infection, the relevance of more in-depth insight into ciprofibrate’s crystal structure to determine its fundamental properties and the possibility to use copolymer encapsulation as a strategy to improve its biodistribution becomes clear.

Herein, we present the crystal structure of CIP in solid state, solved using powder X-ray diffraction data. The drug was also encapsulated in both P123/F127 Pluronic and PEO_113_-*b*-PCL_118_ micelles for comparison via nanoprecipitation technique in water and evaluated in different solvent concentrations. We also propose the best condition for encapsulation for this system and demonstrate the fundamental physicochemical properties of the NPs (size distribution, surface charge, surface morphology). Furthermore, in vitro results and mathematical models for the drug release in buffer solution are presented.

## 2. Materials and Methods

CIP was obtained as a courtesy of DEINFAR (Laboratório de Desenvolvimento e Inovação Farmacotécnica) from the Faculty of Pharmaceutical Sciences (University of São Paulo, São Paulo, Brazil). PEO_113_-*b*-PCL_118_ blocks (Mw = 18,500 g mol^−1^) were purchased from Polymer Source, Inc. (Dorval, QC, Canada). Pluronic F127 (Mw = 12,600 g mol^−1^), Pluronic P123 (Mw = 5750 g mol^−1^), ethanol, acetone, PBS and Tween 20 were purchased from Sigma-Aldrich (São Paulo, Brazil).

### 2.1. Preparation of CIP-Loaded Nanoparticles

The polymeric micelles were produced by nanoprecipitation (as schematized in Figure 1) from stock polymer/drug organic solutions prepared in ethanol. For Pluronic nanoparticles, first, P123 and F127 blocks were weighed and dissolved in ethanol (EtOH) (maintaining a 2:1 *w*/*w* fixed molar ratio of P123 and F127). The polymer concentration was fixed at 10 mg mL^−1^ as proposed by Sortini et al. [21]. This solution was stabilized via sonication for approximately 10 min until it became visually transparent. The organic solution was transferred to a syringe and afterward added dropwise into 5 mL of water solution and then removed by evaporation. For the PEO_113_-*b*-PCL_118_ nanoparticles, acetone was used to completely dissolve the polymeric chains [6,22]. For the CIP-loaded micelles, CIP was weighed and solubilized in ethanol according to the desired feeding and was mixed with the polymeric organic solution before the addition to the aqueous phase. 

### 2.2. Particle Size and Morphology of CIP-Loaded Nanoparticles

The average diameter and size distribution (polydispersity) of the NPs were determined via Dynamic Light Scattering (DLS) and Static Light Scattering (SLS). Samples were loaded into test tubes (10 μL) and diluted in 1 mL of distilled water. Measurements were performed using ALV/CGS-3 platform-based goniometer system (ALV GmbH, Langen, Germany) consisting of a polarized HeNe laser (22 mW) operating at a wavelength λ = 633 nm, an ALV 7004 digital correlator, and a pair of pseudocorrelation APD detectors operating in a crusade mode. The data were collected and further averaged using ALV Correlator Control software. The polydispersity was estimated using the cumulant analysis of the autocorrelation functions measured at 90°. The temporal correlation functions were analyzed using the REPES algorithm (incorporated into the ALV Correlator program) to confirm the monomodal distribution of NPs. The autocorrelation functions reported are based on three independent runs of 60 s counting time for each sample. 

The NPs’ surface charges were obtained via Electrophoretic Light Scattering (ELS) tests. Samples were added into cuvettes (10 µL), placed into the apparatus, and exposed to the laser beam. Experiments were carried out using a Zetasizer Nano-ZS ZEN3600 instrument (Malvern Instruments, Worcestershire, UK). The electrophoretic mobility (µe) was calculated through the Smoluchowski approximation. Each zeta-potential value reported is an average of 3 independent measurements with repeatability of ±2%. 

### 2.3. X-ray Diffraction Analysis

CIP’s crystal structure was determined using Powder X-ray Diffraction (PXRD) data. Moreover, to the best of our knowledge, this is the first time it is reported. The method employed to solve the CIP’s crystal structure is well described in the literature [6,23]. The sample was hand-ground in an agate mortar and loaded between two cellulose acetate foils (0.014 mm) in a spinning sample holder. Powder X-ray diffraction data were collected utilizing a transmission mode copper source, filtered by a germanium monochromator (111). Diffraction intensities were collected by a linear detector Dectris Mythen 1K (Baden-Daettwil, Switzerland) with 0.015° step and integration time of 60 s at every 1.05°. The experiment used a STADI-P (Stoe, Darmstadt, Germany) powder diffractometer available at the Laboratory of Crystallography and Structural Characterization of Materials (LCCEM). 

### 2.4. In Vitro Drug Release Characteristics of CIP-Loaded Nanoparticles

To measure the CIP release from the NPs and to compare the stabilities of both Pluronic and PEO_113_-*b*-PCL_118_, samples with 5 mg mL^−1^ containing 10% (*w*/*w*) and 20% (*w*/*w*) CIP were diluted in PBS pH 7.4 and placed in a dialysis bag (MWCO: 3.500–5.000 Da, Spectra/Por), which was dialyzed against 500 mL of PBS pH 7.4 containing 0.4% (*w*/*v*) Tween 20 at 37 °C for 48 h, under constant magnetic stirring. Aliquots of 50 µL were taken from the dialysis bag at increasing time intervals and afterward diluted 10 times in ethanol and measured through UV-Vis spectroscopy technique using a Cary 50 UV-Vis spectrophotometer (Varian, Inc., Crawley, UK). First, CIP’s analytical calibration curve in EtOH with a linear response in the range 0.0001–0.05 mg mL^−1^ was recorded and used to determine CIP contents. A sample containing empty NPs in EtOH was also measured as a blank sample for comparison. Then, samples were added into cuvettes (10 µL), diluted in EtOH, and placed into the equipment. Dilution proportions varied according to the different investigations, as some samples were highly concentrated and visually turbid, which may affect the results. 

To describe the drug dissolution as a function of time, the drug release profile data were submitted to quantitative analysis, fitted to several kinetic release models. Statistical analysis was performed and indicated the models that best demonstrate the CIP’s release mechanism for both polymeric matrices.

### 2.5. Small-Angle X-ray Scattering 

Small-Angle X-ray Scattering (SAXS) experiments were performed at the beamline B21 of the Diamond Light Source (Didcot, UK) [23,24]. Samples were loaded into quartz capillaries by the Arinax liquid-handling robot and exposed for 1 s, acquiring 20 frames. The wavelength was 0.95 Å, and the camera length was 3.71 m. Modeling to a spherical shell model was done using SASfit. It is worth noting that the samples were stored in a fridge at a range of 4–8 °C for three months before the SAXS experiment.

## 3. Results

### 3.1. Characterization of CIP-Loaded Nanoparticles 

**Figure 2:**
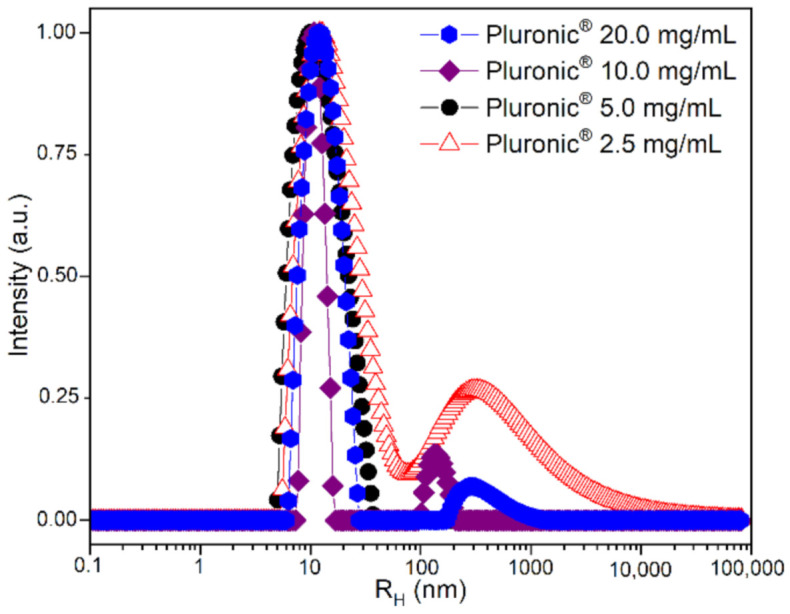
Size distribution of Pluronic micelles measured at 90° by Dynamic Light Scattering.

Table 1 presents general physicochemical aspects of the NPs: their hydrodynamic and gyration radius, polydispersity, and charge, for samples prepared under different concentrations (20–2.5 mg mL^−1^). Figure 2 is a plot obtained from DLS measurements and shows different size distribution profiles for each concentration.

Subsequently, samples were prepared with different CIP amounts and characterized by UV-Vis to determine drug loading content (DLC) and encapsulation efficiency (EE). Results shown in Table 2 indicate a remarkable high efficiency (96%) for the Pluronic micelles prepared with 10% (*w*/*w*) CIP. 

Considering the best values for drug encapsulation in Table 2 (samples that presented more efficient CIP uptake), a new set of NPs was synthesized using the block copolymer PEO_113_-*b*-PCL_118_. Table 3 summarizes aspects such as size, charge, and drug loading for these systems. Table 4 shows the DLC and EE indexes for PEO_113_-*b*-PCL_118_ micelles.

### 3.2. X-ray Diffraction Analysis of CIP

**Figure 3:**
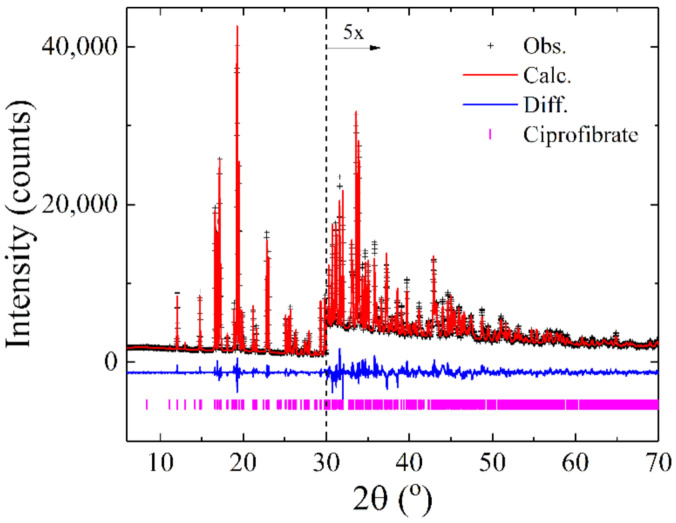
Rietveld plot for CIP showing the excellent agreement between the experimental data (black crosses) and the calculated profile (red line). The blue line displays the difference between observed and calculated data. The magenta vertical bars at the bottom represent the Bragg peaks’ positions. The region from 30 to 70° in 2θ is magnified 5 times to clarify the good agreement between observed and calculated data in higher angles.

**Figure 4:**
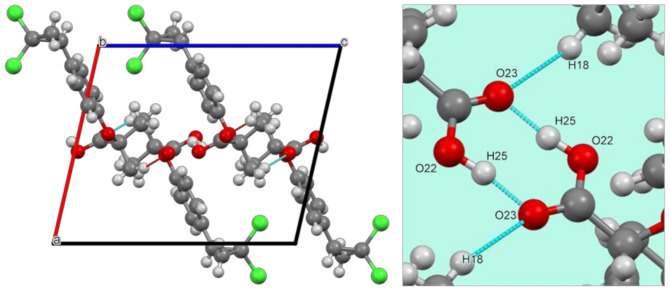
CIP’s crystal structure forms a network of molecular aggregates along the b-axis, with four formula units per unit cell (Z = 4). Hydrogen bonds (cyan lines) are shown on the right side in an enlarged region of the unit cell. Atom color code: red = oxygen (O); green = chloride (Cl), grey = carbon (C); light grey = hydrogen (H).

CIP powder diffraction data were collected to characterize this active pharmaceutical ingredient in the solid state. Figure 3 shows the PXRD diffraction pattern of the pure CIP sample and its final Rietveld refinement, and Table 5 summarizes the complete crystallographic information, deposited in the Cambridge Crystallographic Data Centre under the ID 2097980. Figure 4 shows CIP’s crystal structure. Further details on atom coordinates, bond lengths, and angles can be found in the Supplementary Materials in Tables S1–S4.

### 3.3. Small-Angle X-ray Scattering Analysis of Pluronic and PEO_113_-b-PCL_118_ Nanoparticles’ Structure

**Figure 5:**
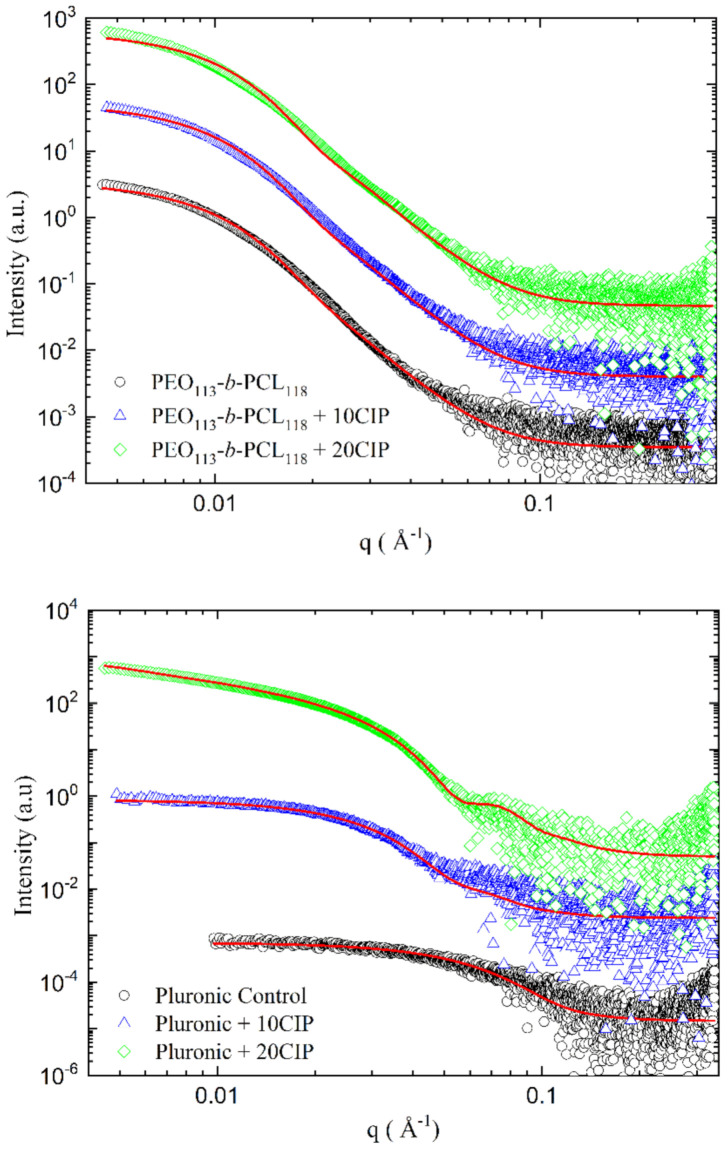
SAXS curves for PEO_113_-*b*-PCL_118_ and Pluronic nanoparticles with different concentrations of CIP in PEO_113_-*b*-PCL_118_ samples (**top**) and Pluronic samples (**bottom**). The fits of data are displayed in red. Details of fits are found in the Supplementary Materials (Tables S5 and S6). Scattering curves have been scaled for the sake of clarity.

Figure 5 shows the data for PEO_113_-*b*-PCL_118_ (top) and Pluronic (bottom) nanoparticles for unloaded and loaded (10 and 20 wt% CIP) samples, fitted to a spherical shell model using SASfit. An exception was made for the Pluronic 20 wt% CIP sample, which was better fitted to a long cylinder. Further details of fits can be found in the Supplementary Materials (Tables S5–S7).

### 3.4. In Vitro Release Profile of CIP from Pluronic and PEO_113_-b-PCL_118_ Nanoparticles

**Figure 6:**
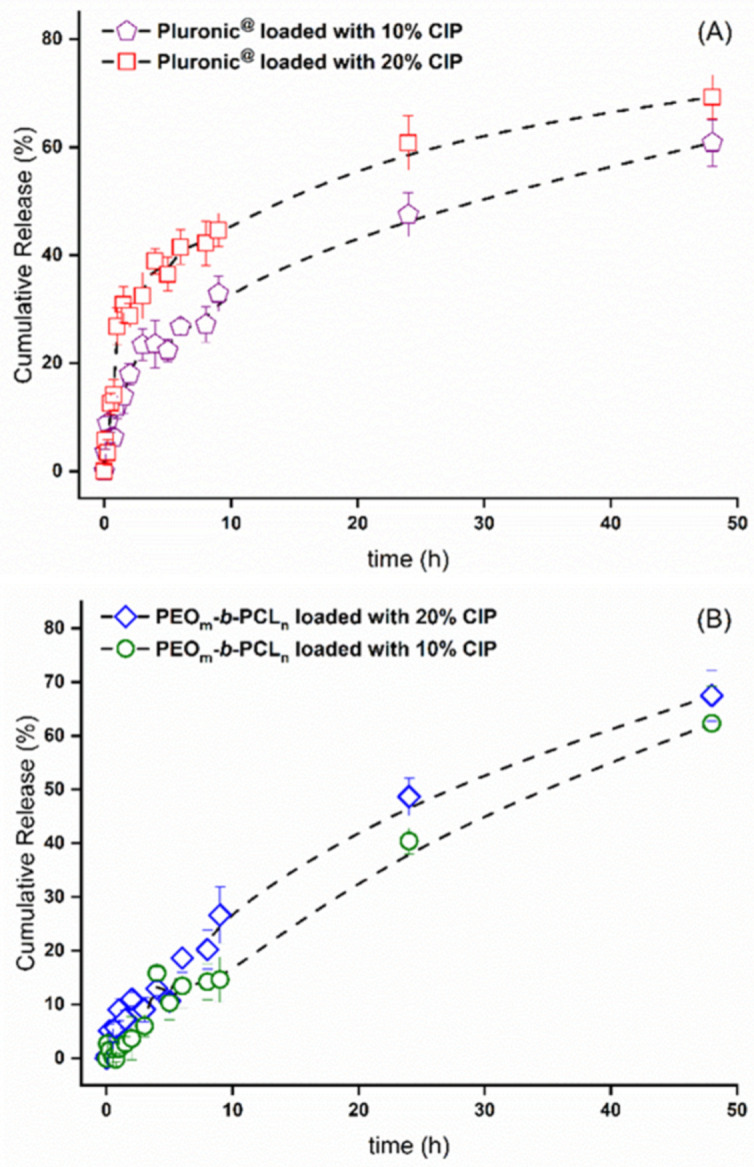
CIP release profile in best encapsulation conditions with (**A**) Pluronic P123/F127 and (**B**) PEO_113_-*b*-PCL_118_ micelles. Error bars indicate the standard deviation of three replicates.

In vitro release was performed for best encapsulation conditions (10% and 20% CIP) using the two polymeric matrices to analyze their influence on the drug release profile. Samples were collected during a 48 h experiment and analyzed via UV-Vis spectrophotometry. Figure 6 shows the release profiles over time obtained for Pluronic (top) and PEO_113_-*b*-PCL_118_ (bottom).

### 3.5. Release Mechanisms for Pluronic and PEO_113_-b-PCL_118_ Nanoparticles

**Figure 7:**
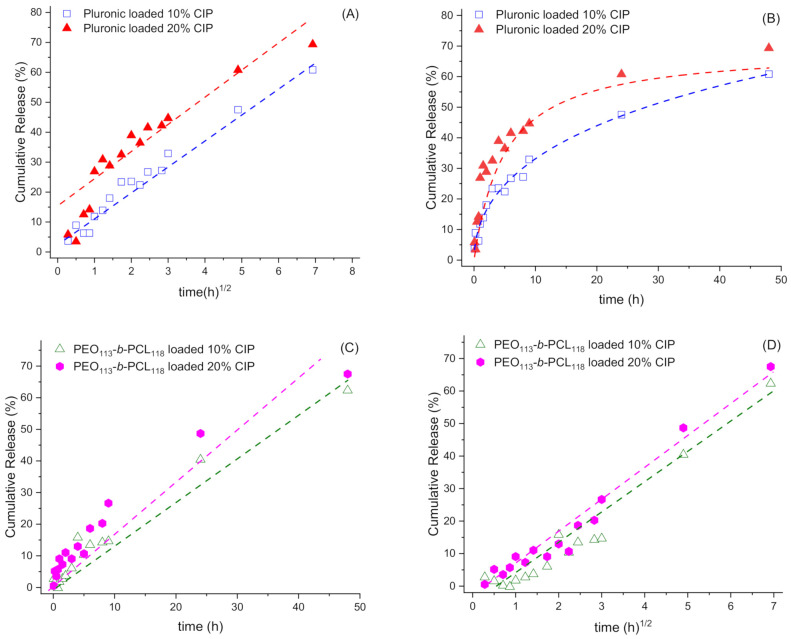
Model fitting using Higuchi (**A**,**D**), Korsmeyer–Peppas (**B**), and zero-order (**C**) approaches based on data given in Figure 6 for Pluronic and PEO_113_-*b*-PCL_118_ nanoparticles loaded with ciprofibrate.

Table 6 presents the result of a statistical analysis of the last release profile data of Pluronic P123/F127 and PEO_113_-*b*-PCL_118_ CIP-loaded nanoparticles, utilizing the linear regression model—except for the Korsmeyer–Peppas model, which was fitted using a polymeric regression—and involving the correlation coefficient (*R*). *R*-values closest to 1 describe a better release mechanism [25]. Figure 7 shows plots of cumulative release data of each of the loaded samples of both polymers over time, fitted to their respective best models. The kinetic constant values of these models are presented in Table 7. 

## 4. Discussion

### 4.1. Physicochemical Aspects of CIP-Loaded Nanoparticles 

Table 1 summarizes the influence of the dispersed system’s concentration (polymeric solution with drug) in the NP aggregation process. No significant differences were observed for the NPs’ diameter and ζ-potential, responsible for their dispersive media stability. It remains valid except for the 2.5 mg mL^−1^ of Pluronic-containing sample, which presented a much larger diameter, higher polydispersity, and a slightly more negative surface charge. It is possible to see in Figure 2 that when the polymeric concentration drops severely, the dilution effect leads to aggregates, increasing size and PDI.

Table 1 also shows the structure-sensitive parameter (*ρ*) values, obtained from DLS and SLS measurements, which could indicate the spherical shape objects. This parameter provides information on the shape, inner structure, and conformation of scattering objects. For this type of assembling, *ρ* is dependent on the inner structure and compactness [26], being close to 0.775 for compact spheres, ~0.8–0.9 for block copolymer micelles due to solvation phenomena, and ~1.0 for hollow spheres and vesicles [27]. Thus, the reported values in Table 1 suggest that the micelles are spherical but highly swollen by water. The 5 mg mL^−1^ polymeric final concentration was selected for the CIP encapsulation study as this condition provides nanoparticles with good features and a concentration of F127 above the critical micelle concentration (CMC) at room temperature (0.357 mM) [28]. Hence, this was the chosen condition for the CIP encapsulation study.

Subsequently, considering the final polymeric concentration in water, it was observed that samples with higher drug concentrations induced sedimentation of drug crystals on the recipient’s bottom after the synthesis. Values shown in Table 2 demonstrate that features such as size and ζ-potential remained the same average from the previous samples (Table 1). However, it is possible to see that for increasing CIP-loading values, there is a decrease in ζ-potential (except for the blank sample). Thus, samples with more efficient CIP uptake corresponded to 10% and 20% of drug amount.

When considered these selected systems produced with PEO_113_-*b*-PCL_118_ micelles (Table 3), it is worth noting that the average size (34 nm) was more significant than for the same Pluronic system (Table 1). For the concentration of 2.5 mg mL^−1^, PEO_113_-*b*-PCL_118_ samples did not show the same high PDI, although this may be observed for the blank sample. The lowest PDI value was obtained for the 20% (*w*/*w*) CIP condition. These polymers present different molar weight distributions and different CMCs; therefore, these distinct aggregation behaviors are expected. 

There is also a reduction in the negative surface charge with increasing CIP loading, probably due to the inversely proportional interaction between PCL chains and the drug. Previous simulations indicate the formation of H-bonds between PCL blocks and hydrophobic drugs [29]; thus, as the CIP amount increases inside the micelle, there is more interaction with PCL chains and consequently less PCL available for interaction with the outer media, reducing the ζ-potential. Additionally, *ρ* factors reached close values compared to the theoretical reference of 0.775 [27], indicating the presence of hard spheres, showing a different hydration profile than Pluronic micelles.

It is essential to highlight that higher CIP ratios were tested; however, CIP’s solubility was limited in acetone. According to data in Table 4, the encapsulation efficiency reaches values closer to 100% compared to previous best Pluronic formulations (Table 2), indicating higher efficiency of the PEO_113_-*b*-PCL_118_ spheres. The DLC values were remarkably similar to those obtained for Pluronic NPs, showing that the produced particles’ size could be a limiting feature related to the final drug amount in the formulation.

### 4.2. CIP’s Crystallographic Characterization

The structural characterization of small drugs such as ciprofibrate is essential to understand how biological activity relates to the physicochemical features. Several drugs are manufactured as crystalline powders (including CIP). Depending on the chemical structure’s degrees of freedom, it might form polymorphs—the ability of a material to exist in two or more crystalline forms with different arrangements or conformations in the crystal lattice—thus changing the crystal structure and, consequently, its properties.

As reported in Table 5, CIP crystallized under a monoclinic crystal system with space group P2_1/c_ and unit cell parameters a = 10.7646(3) Å, b = 10.2368(3) Å, c = 12.8079(4) Å, β = 102.933(2)°, and V = 1375.56(7) Å3. Its structure comprises four formula units per unit cell (Z = 4), accommodating one molecule in the asymmetric unit (Z′ = 1), as shown in Figure 4. Within the unit cell, the molecules are held together by weak hydrogen bonds (or nonclassical) between atoms C(10)–H(18)···O(23), the distances of which are D–H = 0.97(2) Å, H···A = 2.49(2) Å, D···A = 3.41(2) Å, and D–H···A = 159(1)°, and by an intramolecular classical hydrogen bond O(22)–H(25)···O(23) (D–H = 0.993(5) Å, H···A = 1.584(5) Å, D···A = 2.571(5) Å, and D–H···A = 171.8(4)), where “D” and “A” are hydrogen donor and acceptor, respectively, as represented by cyan lines in Figure 4.

### 4.3. Structure of Pluronic and PEO_113_-b-PCL_118_ Nanoparticles

Small-Angle X-ray Scattering (SAXS) is a powerful technique to determine the structural properties of nanoparticles in solution and give their average shape. This gives information on how these nanoparticles may behave in vivo as drug delivery molecules. 

For unloaded PEO_113_-*b*-PCL_118_, SAXS data show polydisperse spheres with an average radius of 12.2 nm (Figure 5, top). This finding agrees with the DLS data, which show a polydisperse distribution of radii. The radii increase to 12.5 nm at 10 wt% CIP and 12.6 nm at 20 wt% CIP upon addition of CIP. Moreover, there is a decrease in the scattering intensity of the core, indicating that CIP is loaded into it. 

For the Pluronic nanoparticles (Figure 5, bottom), a spherical shell was fitted to the unloaded sample and the sample with 10% CIP. Empty Pluronic shows polydisperse spheres with a radius of 2.3 nm, which swells to 5.7 nm upon the addition of 10 wt% CIP. Moreover, the scattering density of the core decreases, implying the loading of the drug. For 20 wt% CIP, the decay of *q*^−1^ in the low-*q* data region implied cylindrical particles. These data were fitted to a long cylinder with a radius of 6.5 nm, implying that the spherical nanoparticles are less stable and may aggregate at this concentration of CIP. It may be inferred that, as these samples were submitted to low-temperature long-term storage, this may have influenced their morphological structure.

### 4.4. In Vitro Release Profile of CIP from Pluronic and PEO_113_-b-PCL_118_ Nanoparticles

The data displayed in Figure 6 indicate a faster release for the mixed Pluronic NPs than for the PEO_113_-*b*-PCL_118_ system. For CIP 20% condition (red squares), nearly 45% of the drug content was released in the first 9 h, while 33% was released from CIP 10% condition (purple pentagons).

For the PEO_113_-*b*-PCL_118_ system, a more consistent release was observed; for the CIP 20% condition, only 25% of the drug was released after 9 h, and for the CIP 10% sample, 16% of the drug was released after 9 h. The data evidenced that nearly 60–70% of the loaded CIP is released within two days and—based on previous studies of our research group with PCL copolymers—the remaining is slowly released within the next day, or it is adsorbed into the dialysis bag [18]. 

This quick drug release in the first hours of in vitro experiments agrees with previous studies concerning mixed Pluronic formulations [21,30,31], although, in this study, a slower profile was obtained. This kinetic may be altered due to several conditions. First, the experiment was conducted at a temperature of 37 °C, in which the F127 is most probably in its gelation form due to its sensitive CMT [32], which tends to release the encapsulated drugs faster. As previously demonstrated [30], mixed formulations of F127 and more hydrophobic Pluronic chains can control the hydrogel formation and increase the release interval, contributing to a more stable composition. This aspect is mainly observed for hydrophobic drugs. The addition of hydrophilic F127 to the composition increases the PEO chain lengths and, consequently, the amount of water in the micelle, leading to so-called hydrophilic channels [21]. Finally, the encapsulated drug solubility may also alter the hydrogel state and, as previously reported, lower the gelification temperature in case of hydrophobic behavior. The release time increment is probably due to the presence of P123 in a higher proportion than F127 in the micelles. However, the temperature and hydrophobicity of the CIP may still contribute to a rapid release.

### 4.5. Release Mechanisms for Pluronic and PEO_113_-b-PCL_118_ Nanoparticles

Considering the obtained release profiles (Figure 6) and the different kinetic (Table 7) and physicochemical features of the block copolymers (Table 1, Table 2, Table 3 and Table 4), it is essential to dig out the associated release mechanism for the produced NPs. In the literature, four different drug release mechanisms are known: (1) drug desorption, (2) drug diffusion, (3) erosion, and (4) combined erosion–diffusion. Accordingly, the CIP concentration values obtained via UV-Vis spectroscopy were later fitted to selected mathematical models to comprehend the release mechanisms involved in both polymeric systems.

It is worth noting in Table 6 that, for Pluronic systems, the Higuchi model presents the highest *R*-values, indicating the release mechanism is diffusional through the micelle, which predicts a rapid release of the drug [25], in agreement with previous studies [30]. This model is a time-dependent linear square root that obeys Fick’s diffusion law; therefore, the release is directly proportional to the surface area and the concentration difference and inversely proportional to the membrane’s thickness. Moreover, it is possible to notice that the Korsmeyer–Peppas *R*-values are also higher for Pluronic systems, reinforcing the diffusion as a suitable proposal for the release mechanism. 

According to Bruschi M. [33], for spherical particles, the release exponent *n* is expected to be 0.43—precisely what was achieved for Pluronic micelles with 10% CIP (Table 7). It is a good approximation for the Pluronic sample with 20% CIP, confirming previous results regarding NPs’ morphology. Additionally, this *n* value also indicates the dominating release mechanism: for *n* < 0.45, liberation occurs by Fickian diffusion [25], suggesting that Higuchi’s model is the preferential form of drug release. However, it is important to highlight that this model is suitable for these drug concentrations. The interaction between drug and polymer and the total volume of the encapsulated drug may alter the release mechanism. 

As for the PEO_113_-*b*-PCL_118_ micelles, both zero-order and Higuchi models describe the release mechanism (Table 6 and Figure 7C,D). According to Costa et al. [25], the zero-order model describes systems that do not disaggregate with time and promote a slower, linear dissolution that does not depend on the drug’s concentration. These are ideal drug delivery systems, as they sustain a prolonged action.

## 5. Conclusions

It was possible to characterize CIP’s crystal structure through this study, which has never been reported in the specific literature before. It crystallizes in a monoclinic crystal system in its solid-state, a pattern maintained due to strong H bonds between the –OH terminations of the molecules. We have also proposed a synthesis method and two polymeric matrices to encapsulate CIP and improve its solubility—a mixed proportion of Pluronic P123/F127 and a matrix composed of PEO_113_-*b*-PCL_118_. These polymers are already well established as biopolymers but have not been tested yet with CIP mainly. Results indicate that both systems produced micelles with suitable physicochemical characteristics, such as small size and relatively neutral zeta potential (from −10 to +10 mV), with great potential to enhance delivery efficiency in the human body [34]. The synthesis method was also demonstrated to be suitable, as repeated productions led to similar samples with minimum deviations. Besides, polydispersity values for the samples were very low, ideal for a stable solution. 

As for the shape of the NPs, morphological characteristics demonstrated good agreement among diverse techniques such as DLS, SLS, SAXS, and statistical analysis using in vitro tests results after UV-Vis spectrophotometry for both systems. The SAXS profiles corroborate the spherical shape expected by combining the DLS/SLS measurements for both polymeric systems, except for the Pluronic micelles with 20 wt% of CIP loading, which presented a morphological change, as discussed adequately in Section 4.3. The small values of the gyration radius also agree with the hydrodynamic radius. Moreover, comparing the scattering densities, results indicate that the drug was incorporated in the nucleus of the polymeric nanoparticles, as we expected.

In vitro release experiments also have indicated that the drug remains in the system for up to 48 h. Although both polymeric systems have shown very similar physicochemical characteristics, PEO_113_-*b*-PCL_118_ micelles showed zero-order release kinetics in in vitro drug release tests. Statistical analyses also indicated that the proposed CIP-loaded mixed Pluronic system has a release mechanism based on Fickian diffusion, which is independent of the amount of CIP encapsulated. In conclusion, concerning the NPs’ physicochemical stability over time, this study proposes that CIP quantities up to 20% (*w*/*w*) can be encapsulated preferentially in PEO_113_-*b*-PCL_118_ micelles as an alternative to increase its hydrophilicity and, therefore, its release time. Considering the recent advances in nanomedicine and the continued efforts to improve drug delivery techniques in order to provide targeted release of drugs, hydrophobicity, and bioavailability [7,35,36], this study represents an initial step to understand both CIP’s physicochemical properties and the proposed nanoparticle system of encapsulation. The present work may also contribute to studies aiming to better comprehend the mechanism of action of the fibrate class drugs in diminishing lipid levels in the human body and, beyond that, to the rising number of studies correlating the presence of dyslipidemia condition with more severe forms of COVID-19 infection [37,38,39]. Therefore, further studies can be conducted for possible improvements in ciprofibrate’s pharmaceutical applications.

## Figures and Tables

**Table 1:** Results for DLS and ELS evaluation for different CIP-loaded Pluronic micelle concentrations ^†^ in water.

Pluronic Concentration (mg mL^−1^)	*R*_H_ (nm)	PDI	ζ-Potential (mV)	*R*_G_ (nm)	$\rho =\frac{R_{G}}{R_{H}}$
20.0	13 ± 3	0.22	−4 ± 1	-	-
10.0	11 ± 2	0.19	−4.3 ± 0.6	14.9 ± 0.5	1.3
5.0	12 ± 2	0.18	−5.9 ± 0.5	16.4 ± 0.5	1.3
2.5	52 ± 12	0.33	−9 ± 3	-	-

^†^ The amount of drug remained fixed as 10% of the total amount of Pluronic (*w*/*w*); PDI stands for the polydispersity of NPs; *R*_H_ is the hydrodynamic radii of each sample in terms of mass distribution; *R*_G_ is the gyration radii of each sample; *ρ* is the structure-sensitive parameter.

**Table 2:** Results for drug content evaluation through UV-Vis spectroscopy for different micelle concentrations in water.

Pluronic Conc. (mg mL^−1^)	Drug Amount (*w*/*w*)	*R*_H_ (nm)	PDI	ζ-Potential (mV)	Amount of Drug by UV (mg mL^−1^)	EE (%)	DLC (%)
5.0	-	12 ± 2	0.21	−4.7 ± 0.9	-	-	-
5.0	10%	10 ± 2	0.20	−7 ± 1	0.48 ± 0.03	96	8.8
5.0	20%	13 ± 2	0.16	−7.3 ± 0.8	0.51 ± 0.02	51	9.3
5.0	30%	14 ± 2	0.18	−5.9 ± 0.7	0.49 ± 0.04	32.6	8.9
5.0	40%	13 ± 2	0.22	−5 ± 1	0.24 ± 0.02	12	4.6

**Table 3:** Results for DLS, ELS, and drug content evaluation for PEO_113_-PCL_118_ micelles with the best CIP concentrations.

PEO/PCL Conc. (mg mL^−1^)	Drug Amount (*w*/*w*)	*R*_H_ (nm)	PDI	ζ-Potential (mV)	*R*_G_ (nm)	$\rho =\frac{R_{G}}{R_{H}}$
2.5	-	34 ± 1	0.38	−22 ± 2	-	-
2.5	10%	41 ± 1	0.12	−12 ± 1	33.5 ± 0.5	0.82
2.5	20%	41.8 ± 0.9	0.08	−9.5 ± 0.7	32.6 ± 0.5	0.78

**Table 4:** Results for drug content evaluation using UV-Vis spectroscopy for PEO_113_-*b*-PCL_118_ micelles with the best CIP concentrations.

Total Polymer (mg)	Total CIP (mg)	CIP Amount (*w*/*w*)	Theoretical CIP Final Conc. (mg mL^−1^)	Amount of Drug by UV (mg mL^−1^)	EE (%)	DLC (%)
5.0 *	-	-	-	-	-	-
5.0	0.5	10%	0.25	0.24 ± 0.01	98	8.7
5.0	1.0	20%	0.50	0.23 ± 0.02	96	8.4

* Control sample.

**Table 5:** Crystal structure of CIP determined via PXRD and statistical information of the Rietveld refinement.

Crystal System	Monoclinic
Space group	*P*2_1_/*c*
a; b; c (Å)	10.7646(3); 10.2368(3); 12.8079(4)
β (°)	102.933(2)
Volume (A^3^)	1375.56(7)
Z; Z′	4; 1
*R*_exp_ (%)	2.637
*R*_wp_ (%)	5.746
*R*_Bragg_ (%)	2.984
χ^2^	2.179

**Table 6:** *R*-values for different drug release model fittings.

Polymeric Matrix	Drug/Polymer Ratio	Zero Order	First Order	Higuchi	Hixson–Crowell	Korsmeyer–Peppas
P123/F127	10%	0.91247	0.70178	**0.98544**	0.78473	**0.96861**
P123/F127	20%	0.80424	0.55803	**0.93048**	0.64722	**0.91804**
PEO/PCL	10%	**0.98031**	0.70064	**0.97411**	0.76204	0.82982
PEO/PCL	20%	**0.97149**	0.69793	**0.98321**	0.84923	0.95231

Bold values indicate *R*-coefficients closest to 1.

**Table 7:** Release kinetic constants obtained from statistical analysis.

Polymeric Matrix	Drug/Polymer Ratio	Zero Order	Higuchi	Korsmeyer–Peppas	
		K_0_ (%h^−1^)	K_H_ (%h^−1/2^)	K_KP_ (%h^−n^)	*N*
P123/F127	10%	-	8.76	0.12	0.43
P123/F127	20%	-	9.73	0.20	0.38
PEO/PCL	10%	1.32	9.39	-	-
PEO/PCL	20%	1.39	10.09	-	-

## Data Availability

Ciprofibrate’s crystallographic information framework file is available on Cambridge Crystallographic Data Centre under the ID 2097980.

## Supplementary Materials

The following are available online at https://www.mdpi.com/article/10.3390/polym13183158/s1, Table S1: Final atomic coordinates of all atoms for ciprofibrate crystal structure; Table S2: Bond lengths; Table S3: All angles; Table S4: Torsion angle data; Table S5: Spherical shell data fits; Table S6: Long cylinder fit for Pluronic + 20CIP; Table S7: Radius of gyration and D_max_ (maximum particle dimensions) calculated using the ATSAS software.
