# Supplementary material for: Ciprofibrate-Loaded Nanoparticles Prepared by Nanoprecipitation: Synthesis, Characterization, and Drug Release
Source: Polymers (Basel). 2021 Sep 18;13(18):3158. doi: 10.3390/polym13183158 (PMC8468397; doi:10.3390/polym13183158)
Supplement: Supplementary file 1 [file polymers-13-03158-s001.zip › polymers-1376542-supplementary.pdf]

Ciprofibrate-loaded nanoparticles prepared by nanoprecipitation: synthesis, characterization, and drug release

Supplementary Information

Table S1: Final atomic coordinates of all atoms for ciprofibrate crystal structure.

Atom	x	y	z
C1	0.33144(19)	0.11517(16)	0.0522(2)
O2	0.4471(2)	0.1300(2)	0.1227(2)
C3	0.3050(2)	-0.0094(2)	0.0142(2)
C4	0.2415(2)	0.2100(2)	0.0161(2)
C5	0.48905(16)	0.24937(17)	0.17598(14)
C6	0.1917(2)	-0.0376(2)	-0.0564(2)
C8	0.1306(2)	0.1814(2)	-0.0537(2)
C10	0.6210(3)	0.2320(5)	0.2496(3)
C11	0.3940(3)	0.2819(5)	0.2461(3)
C12	0.4803(2)	0.36056(17)	0.09599(13)
C13	0.10235(18)	0.05846(17)	-0.09102(18)
O22	0.5324(3)	0.3313(4)	0.0125(2)
O23	0.4546(6)	0.4741(3)	0.1110(3)
C24	-0.0226(2)	0.02759(19)	-0.16700(15)
C26	-0.0598(3)	-0.1084(2)	-0.2078(2)
C27	-0.03458(16)	-0.0052(2)	-0.28304(12)
Cl31	0.1032(2)	-0.0179(4)	-0.3354(2)
Cl32	-0.1641(2)	0.0584(3)	-0.3786(2)
H7	0.3652(2)	-0.0777(2)	0.0393(2)
H9	0.2569(2)	0.2980(2)	0.0410(2)
H14	0.1748(2)	-0.1259(2)	-0.0800(2)
H15	0.0694(2)	0.2497(2)	-0.0757(2)
H16	0.6809(3)	0.2101(5)	0.2069(3)
H17	0.6455(3)	0.3128(5)	0.2861(3)
H18	0.6179(3)	0.1688(5)	0.3042(3)
H19	0.3925(3)	0.2157(5)	0.2988(3)
H20	0.4175(3)	0.3614(5)	0.2849(3)

H21	0.3085(3)	0.2941(5)	0.2050(3)
H25	0.5379(3)	0.4115(4)	-0.0296(2)
H28	-0.0932(2)	0.0752(2)	-0.1516(2)
H29	-0.1435(3)	-0.1419(2)	-0.2093(2)
H30	-0.0015(3)	-0.1791(2)	-0.1858(2)

Table S2: Bond lengths

Atom 1	Atom 2	Bond Length (Å)
O2	C1	1.372(3)
C3	C1	1.375(3)
C4	C1	1.378(3)
O2	C5	1.426(3)
C6	C3	1.376(3)
C8	C4	1.353(3)
C10	C5	1.528(3)
C11	C5	1.541(4)
C5	C12	1.522(2)
C6	C13	1.379(3)
C8	C13	1.360(3)
O22	C12	1.347(4)
O23	C12	1.223(4)
C13	C24	1.506(3)
C26	C24	1.513(3)
C27	C24	1.500(2)
C26	C27	1.497(3)
C27	Cl31	1.763(3)
C27	Cl32	1.761(3)

Table S3: All angles

Atom 1	Atom 2	Atom 3	Bond Angle (°)
O2	C1	C3	114.9(2)
O2	C1	C4	127.4(2)
C4	C1	C3	117.7(2)

C5	O2	C1	123.6(2)
C6	C3	C1	120.7(2)
C8	C4	C1	121.1(2)
O2	C5	C10	110.2(2)
O2	C5	C11	106.5(2)
O2	C5	C12	111.0(2)
C11	C5	C10	108.2(2)
C10	C5	C12	114.3(2)
C11	C5	C12	106.3(2)
C3	C6	C13	120.8(2)
C4	C8	C13	121.9(2)
O22	C12	C5	113.1(2)
O23	C12	C5	126.3(3)
O22	C12	O23	119.1(3)
C8	C13	C6	117.8(2)
C6	C13	C24	120.9(2)
C8	C13	C24	121.3(2)
C26	C24	C13	123.1(2)
C27	C24	C13	123.6(2)
C26	C24	C27	59.6(1)
C27	C26	C24	59.8(1)
C26	C27	C24	60.7(1)
C24	C27	Cl31	120.0(2)
C24	C27	Cl32	118.9(2)
C26	C27	Cl31	118.8(2)
C26	C27	Cl32	118.7(2)
Cl32	C27	Cl31	111.4(2)

Table S4: Torsion angle data

Atom 1	Atom 2	Atom 3	Atom 4	Torsion Angle (°)
C5	O2	C1	C3	-176.1(2)
C5	O2	C1	C4	4.2(4)

C1	O2	C5	C10	179.3(2)
C1	O2	C5	C11	62.2(3)
C1	O2	C5	C12	-53.1(3)
O2	C5	C12	O22	-48.6(3)
O2	C5	C12	O23	146.0(4)
O22	C12	C5	C10	76.8(3)
O23	C12	C5	C10	-88.7(5)
O22	C12	C5	C11	-164.0(2)
O23	C12	C5	C11	30.6(5)
C26	C24	C13	C6	1.5(3)
C27	C24	C13	C6	-71.4(3)
C26	C24	C13	C8	-178.0(2)
C27	C24	C13	C8	109.0(2)

Table S5: Spherical shell data fits

**Sample**	**R_1 (nm)**	***PD of R_1 (nm)***	***R_2 (nm)***	μ	η	**BG**
PEO_{113}-*b*-PCL_{118}	12.2	6.6	12.0	1	$7.8x10^{-8}$	0.004
PEO_{113}-*b*-PCL_{118} 10 wt% CIP	12.5	6.6	12.0	0.97	$9.3x10^{-8}$	0.004
PEO_{113}-*b*-PCL_{118} 20 wt% CIP	12.6	6.6	12.0	0.91	$1.1x10^{-8}$	0.004
Pluronic	2.3	1	1	1	$6.7x10^{-7}$	0.0001
Pluronic + 10 wt% CIP	5.7	1.9	5.5	0.83	$6.7x10^{-7}$	0.0003

R_1 is the outer radius of the sphere
PD is polydispersity of the outer radius
R_2 is the inner radius of the sphere
η is scattering contrast of the shell
μ is the scattering contrast of the core and BG is the background

Table S6: Long Cylinder fit for Pluronic + 20CIP

**Sample**	**R (nm)**	***PD of R (nm)***	η	**BG**
Pluronic + 20 w% CIP	6.5	1.1	$1.0x10^{-5}$	0.0001

R is the radius of the cylinder
PD is polydispersity of the cylinder
η is scattering contrast of the cylinder
BG is the background

Table S7: Radius of gyration and D_{max} (maximum particle dimensions) calculated using the ATSAS software.

**Sample**	**R_g (nm)**	**D_{max} (nm)**
PEO_{113}-*b*-PCL_{118}	22.2(8.0)	80
PEO_{113}-*b*-PCL_{118} 10 w% CIP	22.2(8.2)	80
PEO_{113}-*b*-PCL_{118} 20 w% CIP	22.9(8.1)	80
Pluronic	3.7(0.3)	14
Pluronic + 10 w% CIP	6.9(0.4)	20
Pluronic + 20 w% CIP	24.8	100
